# Polymorphisms in the Innate Immune *IFIH1* Gene, Frequency of Enterovirus in Monthly Fecal Samples during Infancy, and Islet Autoimmunity

**DOI:** 10.1371/journal.pone.0027781

**Published:** 2011-11-14

**Authors:** Elisabet Witsø, German Tapia, Ondrej Cinek, Flemming Michael Pociot, Lars C. Stene, Kjersti S. Rønningen

**Affiliations:** 1 Norwegian Institute of Public Health, Oslo, Norway; 2 2nd Faculty of Medicine, Charles University Prague, Prague, The Czech Republic; 3 Glostrup Research Institute, Glostrup, Denmark; 4 Oslo University Hospital, Rikshospitalet, Oslo, Norway; South Texas Veterans Health Care System, United States of America

## Abstract

Interferon induced with helicase C domain 1 (IFIH1) senses and initiates antiviral activity against enteroviruses. Genetic variants of *IFIH1*, one common and four rare SNPs have been associated with lower risk for type 1 diabetes. Our aim was to test whether these type 1 diabetes-associated *IFIH1* polymorphisms are associated with the occurrence of enterovirus infection in the gut of healthy children, or influence the lack of association between gut enterovirus infection and islet autoimmunity.

After testing of 46,939 Norwegian newborns, 421 children carrying the high risk genotype for type 1 diabetes (*HLA*-*DR4-DQ8/DR3-DQ2*) as well as 375 children without this genotype were included for monthly fecal collections from 3 to 35 months of age, and genotyped for the *IFIH1* polymorphisms. A total of 7,793 fecal samples were tested for presence of enterovirus RNA using real time reverse transcriptase PCR.

We found no association with frequency of enterovirus in the gut for the common *IFIH1* polymorphism rs1990760, or either of the rare variants of rs35744605, rs35667974, rs35337543, while the enterovirus prevalence marginally differed in samples from the 8 carriers of a rare allele of rs35732034 (26.1%, 18/69 samples) as compared to wild-type homozygotes (12.4%, 955/7724 samples); odds ratio 2.5, p = 0.06. The association was stronger when infections were restricted to those with high viral loads (odds ratio 3.3, 95% CI 1.3–8.4, p = 0.01). The lack of association between enterovirus frequency and islet autoimmunity reported in our previous study was not materially influenced by the *IFIH1* SNPs.

We conclude that the type 1 diabetes-associated *IFIH1* polymorphisms have no, or only minor influence on the occurrence, quantity or duration of enterovirus infection in the gut. Its effect on the risk of diabetes is likely to lie elsewhere in the pathogenic process than in the modification of gut infection.

## Introduction

Enteroviruses (family *Picornaviridae*) cause mostly inapparent or subclinical infections; acute disease may range from minor illness to paralytic disease [1]. Enteroviruses are thought to cross the intestinal epithelium via M-cells [2] and to replicate primarily in the lymphoid tissues of the gut [3], [4].

*IFIH1* (interferon induced with helicase C domain 1), also known as *MDA5* (melanoma differentiation associated gene 5), encodes a cytoplasmic sensor that recognizes certain types of double stranded RNA (dsRNA) molecules, which are commonly produced during the replication of some RNA viruses. Signaling via IFIH1 triggers activation of NF-κB and interferon regulatory pathways, and induces antiviral interferon responses [5]. IFIH1 is triggered by picornaviruses [6], [7], and two recent publications have found *in vivo* effects of IFIH1 in *mda5* knockout mice infected with coxsackievirus [8], [9].

Enteroviruses have been considered as possible environmental triggers or accelerators of islet autoimmunity leading to type 1 diabetes [10]–[12]. The relation seems complex, with partially conflicting results [13]; the research has been going on for more than 30 years now. This hypothesis was greatly sparked when a genome-wide association study of type 1 diabetes identified a significant relation with a common polymorphism in *IFIH1* [14]. This association is now convincingly established (odds ratio around 0.85 for the minor allele) [15]–[19].

Recently, deep sequencing of exons and splice sites in the *IFIH1* gene revealed that lower risk of type 1 diabetes is also associated with two rare variants with a presumed loss of function (nonsynonymous SNPs rs35744605 (E627X) and rs35667974 (I923V)), and with two noncoding variants affecting conserved splice sites (rs35337543 (1641+1G>C)) and rs35732034 (2807+1G>A)), odds ratios of about 0.5–0.7 [20]. The mechanisms relating *IFIH1* polymorphisms to type 1 diabetes, and if enterovirus is involved here, remains unclear and paradoxical. Presumably, variants associated with reduced IFIH1 function would confer the host with a mild antiviral response to enterovirus infection. If so, this would influence the observed association between enterovirus frequency and type 1 diabetes in the population, and failure to account for *IFIH1* SNPs in population studies could be hypothesized to “conceal” relations between enterovirus and islet autoimmunity or type 1 diabetes.

The primary aim of the present study was to assess in healthy children whether the *IFIH1* polymorphisms associated with type 1 diabetes can predict frequency, viral load, or duration of gut infections with enterovirus. We further aimed to test whether our previously reported lack of association between enterovirus and islet autoimmunity was modified by taking these *IFIH1* polymorphisms into account.

## Methods

### Subjects and study design

**Figure 1:**
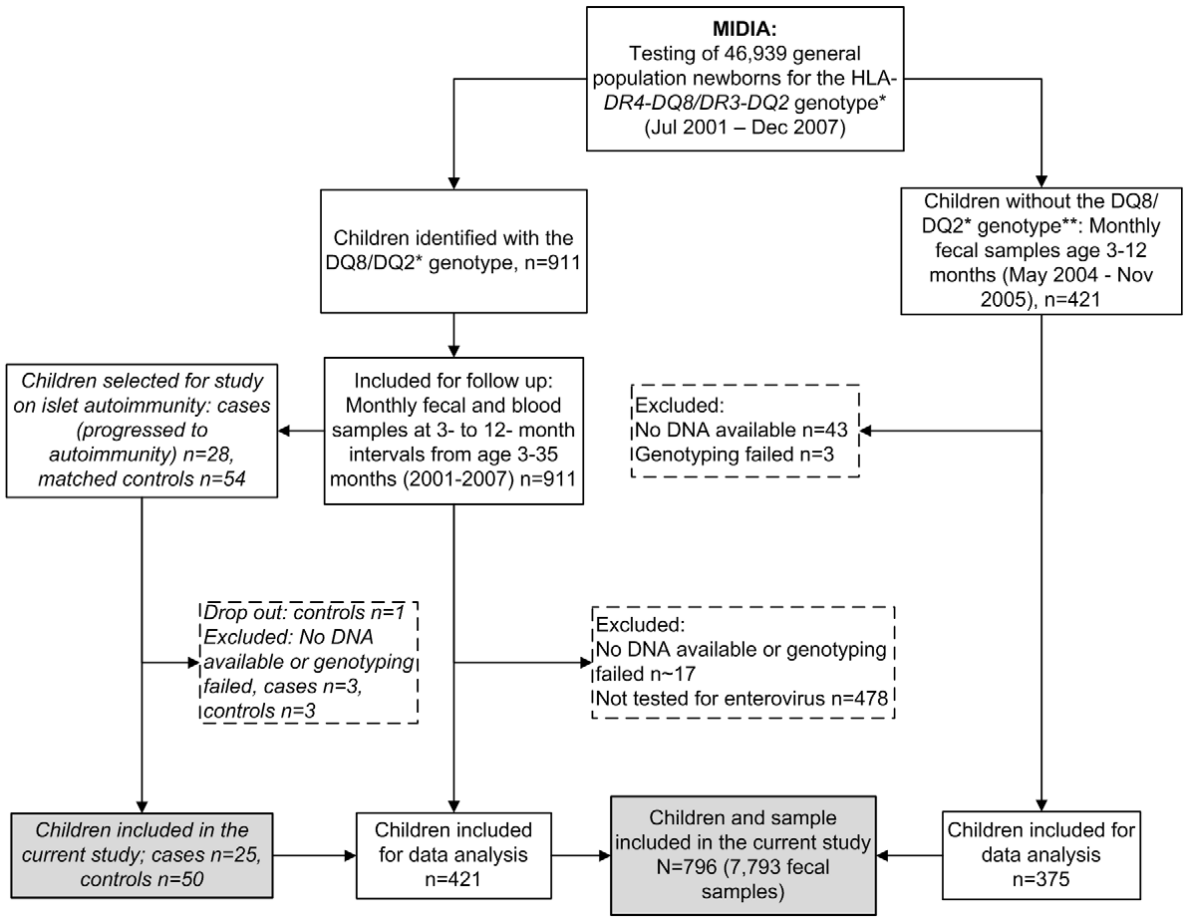
Flow chart illustrating the details of inclusion of children and samples in the current analysis. *HLA-DRB1*04:01-DQA1*03-DQB1*03:02/DRB1*03-DQA1*05-DQB1*02 (*DR4-DQ8/DR3-DQ2*), conferring high risk for type 1 diabetes. **For each child identified with the high-risk genotype, about 1–4 children without the high risk genotype were enrolled from the same county.

The participants were identified and recruited in the MIDIA-study that during 2001–2007 screened 46,939 newborns from the Norwegian general population for the HLA genotype DRB1*04:01-DQA1*03-DQB1*03:02/DRB1*03-DQA1*05-DQB1*02 (DR4-DQ8/DR3-DQ2) which confers high risk for type 1 diabetes [21]. The present analysis is based on nearly 8,000 fecal samples from 421 children with the *HLA DR4-DQ8/DR3-DQ2* genotype and 375 children without this genotype, as described in detail in Figure 1.

In a separate analysis, we studied whether the *IFIH1* polymorphisms modified the risk of type 1 diabetes-related islet autoimmunity among children with the *HLA-DR4-DQ8/DR3-DQ2* genotype (Figure 1). Blood samples from these children drawn at ages 3, 6, 9 and 12 months, and annually thereafter were tested for islet autoantibodies (to insulin, glutamic acid decarboxylase, or the protein phosphatase IA2) using radiobinding assays as previously described [21]. The endpoint of islet autoimmunity was defined as repeated positivity for two or three islet autoantibodies. Controls without islet autoimmunity were matched for the *HLA DR4-DQ8/DR3-DQ2* genotype, date of birth, length of follow-up and county of residence [13] (Figure 1).

### Ethics statement

Written informed consent was provided by participating families. The study was approved by The Regional Committee for Medical Research Ethics (IRB name: “Regional Med Resch Ethics Comm South IRB #2 - South-East A”, IRB00001871) and the Norwegian Data Inspectorate.

### Detection of enterovirus RNA in fecal samples

Fecal samples were tested for enterovirus as described earlier [13]. Nucleic acids were co-purified from supernatants of centrifuged feces, and tested for human enterovirus RNA using an internally controlled quantitative reverse transcriptase real-time PCR with primers and probes described in [22]. The median number of fecal samples tested per child was 9 (range 1–36) and the median age at last fecal sample was 12.1 months (range 2.8–37.5).

### Genotyping of IFIH1 SNPs

Subjects were genotyped for one common *IFIH1* SNP (rs1990760, A946T), and four rare SNPs rs35744605 (E627X), rs35667974 (I923V), rs35732034 (intron 14, 2807+1) and rs35337543 (intron 18, 1641+1). SNP genotyping of rare variant SNPs was performed with the MassARRAY system using iPLEX chemistry (Sequenom, San Diego, CA). The common SNP rs1990760 was genotyped using TaqMan technology with predesigned primer-probe mixes (Applied Biosystems, Foster City, CA, USA). Among children with at least one fecal sample tested for enterovirus (n = 808), genotyping with the MassARRAY system failed in 5–8 DNA samples (<0.1%), and with TaqMan only two samples failed. Duplicate genotyping was performed in 5% randomly chosen samples, with 100% concordance.

### Statistical analysis

The final sample for analysis included 796 children and their 7,793 fecal samples (Figure 1). There was no evidence for deviation from Hardy–Weinberg equilibrium (χ^2^ test) in either of the polymorphisms. Haploview 4.2 was used to assess linkage disequilibria between the alleles of the typed SNPs. Association analyses were performed using logistic regression models with enterovirus infection (yes or no) as the response, individual SNP genotypes as predictors, and a random intercept to account for potential intra-individual correlation (clustering) of infections (xtmelogit in STATA11, StataCorp).

Apart from taking enterovirus positivity as an outcome, in further sensitivity analysis we restricted infections to those with above median quantity of enterovirus RNA among positive samples (defined as high viral load: 10,000 or more virus copies/µl), or to the first enterovirus RNA positive samples among series of two or more consecutively positive samples. Prolonged infection episodes with at least two consecutively positive samples were assessed by excluding other positive samples.

Furthermore, cases of islet autoimmunity and matched controls from our previous publication [13] were compared with respect to enterovirus frequency, and the influence of the *IFIH1* SNPs evaluated. This was modeled similarly as for the *IFIH1* SNP-enterovirus relation, but with autoimmunity status as a covariate and an additional random intercept to specify matched set (three level random intercept model [23]). These analyses were adjusted for, and stratified by the common variants of *IFIH1* SNP rs1990760. The impact of the four rare variant *IFIH1* SNPs on the relation between enterovirus infections and islet autoimmunity could only be evaluated by exclusion of subjects with rare variant of either SNP. Matched case-control data at the individual level were done using conditional logistic regression, with additional adjustment for cumulative number of enterovirus infections before seroconversion for islet autoantibodies for cases, and the corresponding time for matched controls.

In our pre-study power considerations, we used parameters from our previously published pilot studies [22], [24] and the literature [14], [20], and estimated that we could detect odds ratios of at least 1.3 of the common *IFIH1* A946T variant, and odds ratios at about 2–4 for the various rare variants with approximately 80% power.

## Results

### Cohort study of IFIH1 SNPs and enterovirus frequency

The frequencies of enterovirus in fecal samples by genotypes of the five SNPs are shown in Table 1. While neither the common polymorphism rs1990760, nor the rare rs35744605, rs35337543, rs35667974 of *IFIH1* showed any association with presence, quantity or duration of enterovirus RNA in the gut (Table 1 and Table S1), we observed a statistically non-significant tendency towards association for rs35732034 (Table 1). Here the enterovirus prevalence was 26.1% (18/69 samples) among the 8 children carrying the GA genotype, and 12.4% (955/7724 samples) among children with the wild type variant (OR = 2.5, *P* = 0.06).

**Table 1:** Prevalence (%) of enterovirus RNA in fecal samples and frequency of positive samples with a high viral load according to *IFIH1* genotypes.

		Enterovirus prevalence			Samples of high viral load*		
		N = 7,793 (796 children)			N = 7,309 (793 children)		
*IFIH1* SNPs† ‡	Genotypes	Frequency %	OR		Frequency %	OR	
	(n children)	(n samples/n total)	(95% CI)	*P*-value	(n samples/n total)	(95% CI)	*P*-value
rs35337543	GG (788)	12.5 (963/7,677)	1.0 (ref)		6.3 (483/7,676)	1.0 (ref)	
	CG (8)	8.6 (10/116)	0.78 (0.30–2.04)	0.61	5.2 (6/116)	0.89 (0.31–2.51)	0.82
rs35744605	GG (786)	12.5 (964/7,700)	1.0 (ref)		6.3 (483/7,699)	1.0 (ref)	
	GT (10)	9.7 (9/93)	0.90 (0.35–2.37)	0.84	6.5 (6/93)	1.19 (0.43–3.30)	0.74
rs35667974	AA (752)	12.6 (926/7,376)	1.0 (ref)		6.4 (468/7,375)	1.0 (ref)	
	AG (44)	11.3 (47/417)	0.86 (0.55–1.36)	0.53	5.0 (21/417)	0.78 (0.46–1.33)	0.36
rs35732034	GG (788)	12.4 (955/7,724)	1.0 (ref)		6.2 (477/7,723)	1.0 (ref)	
	GA (8)	26.1 (18/69)	2.47 (0.97–6.30)	0.06	17.4 (12/69)	3.35 (1.34–8.38)	0.01
rs1990760	CC (135)	10.6 (130/1,229)	1.0 (ref)	0.72§	5.5 (67/1,128)	1.0 (ref)	0.91§
	CT (386)	13.1 (493/3,756)	1.22 (0.91–1.63)		6.8 (255/3,756)	1.25 (0.89–1.74)	
	TT (275)	12.5 (350/2,808)	1.12 (0.82–1.52)		6.0 (167/2,808)	1.10 (0.77–1.56)	

SNP, single nucleotide polymorphism; OR, odds ratio; CI, confidence interval.

*Excluding enterovirus positive samples with a low-intermediate virus quantity (below 10,000 virus copies/µl enterovirus RNA).

† Location of *IFIH1* SNPs: rs35337543 (intron 18, 1641+1, G>C), rs35744605 (exon 10, E627X, G>T), rs35667974 (exon 14, I923V, A>G), rs35732034 (intron 14, 2807+1, G>A), rs1990760 (exon 14, A946T, T>C).

‡ Reports of functional effects associated with *IFIH1* SNPs: rs35337543 and rs35732034 influences on a putative splice site, rs35744605 is associated with loss of function (ATPase activity, dsRNA binding, truncation of protein), rs35667974 is associated with loss of function (ATPase activity, dsRNA binding), and rs1990760 is not associated with loss of function (reviewed in [29]).

§ Test for trend (1 d.f.).

This suggestive association was strengthened when the analysis was restricted to infections with high viral loads (OR = 3.4, *P* = 0.01, Table 1). The association with high viral load remained significant after adjustment for age, calendar year and season of sample collection (p-values 0.01 to 0.025 and ORs 3.35 to 2.97, data not shown). The association with this rare variant of rs35732034 was also consistent (but not statistically significant) when restricting the analysis to infectious episodes (OR = 1.9, P = 0.06) or to prolonged infections (OR = 2.5, *P* = 0.09) (Table S1). As there were no correlations between the tested SNPs (all r^2^<0.04), the above association is unlikely to be explained by linkage disequilibrium to other known diabetes-associated variants. The association of each *IFIH1* SNP with enterovirus was similar among children with and without the type 1 diabetes-associated *HLA DR4-DQ8/DR3-DQ2* high-risk genotype, respectively (data not shown).

### Nested case control study of islet autoimmunity

We next assessed whether taking the *IFIH1* polymorphisms into account would modify our previously published observation of no association between enterovirus frequency and islet autoimmunity [13]. Adjusting for rs1990760 and excluding the 1 case and 6 controls with a rare variant of rs35744605, rs35667974, rs35732034, or rs35337543 did not influence the results materially (Table 2). There was no significant heterogeneity in the association between enterovirus frequency and islet autoimmunity that would depend on the common *IFIH1* rs1990760 (Table 2). Finally, rs1990760 was not significantly associated with persistent autoimmunity before or after adjusting for the cumulative number of enterovirus infections within a child prior to the development of persistent autoimmunity (Table 2).

**Table 2:** Frequency of enterovirus RNA in faecal samples prior to islet autoimmunity and matched controls and influence of *IFIH1* polymorphisms.

	Cases*	Controls*	OR (95% CI)†	OR (95% CI)†
	(n = 25 subjects)	(n = 50 subjects)	Unadjusted	Adjusted
*EV infection and later development of islet autoimmunity:*				
EV−	282	555	1.00 (reference)	1.00 (reference)
EV+	42 (13.0%)	93 (14.4%)	0.95 (0.58–1.55), P = 0.83	0.99 (0.62–1.60)‡
*The association of IFIH1 rs1990760 polymorphisms with islet autoimmunity:*				
rs1990760 Ala−	9 (36.0%)	20 (40.0%)	1.00 (reference)	1.00 (reference)
rs1990760 Ala+	16 (64.0%)	30 (60.0%)	1.18 (0.44–3.14); P = 0.74	OR = 1.2 (0.44–3.26)§
*EV infection and later development of islet autoimmunity, stratified by IFIH1 rs1990760:*				
EV−, rs199076, Ala−	181 (86.2%)	300 (86.0%)	1.00 (reference)	
EV+, rs199076, Ala−	29 (13.8%)	49 (14.0%)	0.77 (0.34–1.77)	
EV−, rs199076, Ala+	101 (88.6%)	255 (85.3%)	1.00 (reference)	
EV+, rs199076, Ala+	13 (11.4%)	44 (14.7%)	1.04 (0.50–2.15)	P(interaction) = 0.74∥

EV, enterovirus; OR, odds ratio; CI, confidence interval.

*Cases defined as repeated positivity in consecutive blood samples for two or three islet autoantibodies (anti-insulin, anti-GAD, anti-IA2). Controls matched by high risk HLA genotype, date of birth, time of follow-up and county of residence. See reference [13] for more details.

† Estimated from a three level random intercept logistic regression model with enterovirus positivity as dependent variable and case/control status and *IFIH1* SNPs as independent variables. Nested random effects were specified for individuals (samples within individuals) and for matched set (individuals within matched sets of a case and 1–2 controls).

‡ Enterovirus adjusted for *IFIH1* common variant rs1990760 and vice versa. Rare variants could not be adjusted for because of zero observations in one of the comparing groups. Instead subjects/samples with rare variants of these SNPs were excluded from the analysis (1 case, 6 controls; in total 184 samples).

§ Adjusted for the cumulative number of enterovirus infections within a child until persistent autoimmunity develops.

∥ Testing whether the model stratified for Thr/Thr is significantly different from the model stratified for carriers of at least one Ala allele.

## Discussion

We found a potential association between a presumed loss of function variant in the *IFIH1* gene, the rare SNP rs35732034, and a higher frequency of high quantity fecal shedding of enterovirus in infants. Our most important finding however, was the lack or minor influence of the *IFIH1* gene variants on the occurrence, quantity or duration of enterovirus gut infections of healthy infants. Furthermore, taking all these five *IFIH1* polymorphisms into account did not influence our previously reported lack of association between enterovirus frequency and risk of islet autoimmunity in children with the type 1 diabetes susceptibility *HLA-DR4-DQ8/DR3-DQ2* genotype.

### Strengths and limitations

To our knowledge, this is the first study investigating the potential impact of diabetes-associated *IFIH1* polymorphisms on the frequency and viral load of enterovirus in the gut of healthy infants. Strengths of the present study are the very large number of samples analysed, the population based, longitudinal design, and the rather frequent interval of fecal sample collection. As enteroviruses are thought to replicate primarily in the intestine, and the virus is shed for weeks after an infection, our study is likely to cover the majority of enterovirus infections. We did not study systemic spread of enterovirus, as blood samples would have to be extremely frequent in order to systematically cover most infections: enterovirus is usually present in the blood for a much shorter period than in the gut [1].

We are aware of only two previous studies relating *IFIH1* variants to enterovirus RNA in humans, one that investigated the common rs1990760 but which was severely underpowered for genetic association analysis [25] and one relatively large study of young adult patients with established type 1 diabetes (and non-diabetic controls) that investigated two rare type 1 diabetes *IFIH1* variants (rs35744605 and rs35667974) [26]. Both of these studies assayed samples from a single time point from each subject, and may consequently have missed important information on the temporal dimension of the infection. Nevertheless, the rare variant of rs35667974 was slightly more frequent among patients positive for enterovirus RNA in blood compared to enterovirus negative patients [26]. These studies [25], [26], like ours, lack information on enterovirus serotype (genotype). The high number of specific types of enterovirus would require a prohibitively large number of samples for reasonable statistical power for individual serotypes. While we had good power to detect potential associations of small to moderate magnitude (OR<2.0) of the common *IFIH1* SNP our data suggest that the per allele odds ratio is highly unlikely to be greater than about 1.2 (upper 95% CI for per allele trend OR). We cannot exclude weak to moderate effects of the rare variants because of limited power, but the possible existence of such weak effects of rare *IFIH1* variants on enterovirus infections would have limited population impact on the epidemiology of enterovirus infections, if any at all.

### Potential explanations of our main findings

If the suggestive association between the rare A variant of rs35732034 and frequency of high quantity enteroviral infections is true, this indicates a dominant or dose-dependent effect of the predicted interference with splicing of the intron [20]. We can only speculate on the potential mechanisms explaining this phenomenon, while the other established type 1 diabetes associated polymorphisms did not predict enterovirus infections. Predictions based on sequence analysis of the rare variants [20], supported by some experimental functional studies [26]–[28], suggest that the rare variants associated with lower risk of type 1 diabetes are loss of function variants (functional studies are reviewed in [29]). Most such functional in vitro studies are limited to one or a few aspects of function in one or a few cell types taken out of their in vivo context, and perhaps with limited or uncertain sensitivity. We must keep in mind that the endpoint observed in the current study, fecal shedding of enteroviral RNA, is the likely the result of a very complex interaction between the human host and the viral agent and probably activation of a number of signaling pathways in multiple cell types. While there is no obvious reason to suspect that the functional consequences of the rare variant of rs35732034 should be more severe than that of the other three rare variant SNPs studied, our results indicate that one normal allele is sufficient for eliciting a physiological normal antiviral host immune control of enterovirus infections in the gut of otherwise healthy infants, at least for the SNPs not associated with enterovirus frequency. One reason for lack of association could be the involvement of complementary innate antiviral pathways, such as those involving the Toll-like receptors (reviewed in [30]), explaining why IFIH1 was not essential for IFN-production after an enterovirus infection in *mda5* knockout mice [8]. Another potential explanation could be one of the pathogen survival strategies of host immune evasion involving degradation of host IFIH1 signaling molecules [31]. The two groups who have experimentally infected *mda5* knockout mice with coxsackievirus have produced partially conflicting results, but one group showed significantly decreased production of type I IFN, but this was not correlated with increased virus titers [9]. The other group showed that type I IFN levels were not affected, but viruses titers were markedly increased transiently [8].

### Enterovirus infections and type 1 diabetes related islet autoimmunity

The evidence for the involvement of the *enterovirus* genus in type 1 diabetes pathogenesis seem likely, yet the limited numbers of available longitudinal studies of islet autoimmunity where enteroviral RNA have been tested as a risk factor have been inconsistent between populations and sources of the sample [13], [32]–[38]. The evidence seems to be limited to detection in blood or its fractions and predominantly studies done in Finland where serum enterovirus RNA [38] or combined with increases in enterovirus antibodies have been used as indicators of infection [34]–[36]. This is in contrast to studies from other countries (USA and Germany) showing no association [32], [33]. Neither, studies assessing enterovirus RNA in fecal specimens show any association with islet autoimmunity [13], [32], [37].

The *IFIH1* alleles that had been previously associated with reduced type 1 diabetes risk were predicted to be loss of function variants, and may thus confer increased risk for enterovirus infection. We therefore reasoned that our previously published lack of association between enterovirus RNA and islet autoimmunity [13] could be influenced by adjustment for the *IFIH1* SNPs. However, adjusting for *IFIH1* (rs1990760) and the exclusion of children with rare *IFIH1* alleles did not influence the lack of association. A prospective cohort study recently reported lack of association with islet autoantibody development for genotypes of the *IFIH1* SNP rs2111485, a SNP in close proximity and in strong linkage disequilibrium with rs1990760. While this may partially be explained by limited statistical power, their study showed an association with faster progression from autoimmunity to type 1 diabetes [39]. This may hint towards a potential role in a different immunological context, but which would be very demanding to study in humans.

In conclusion, despite the predictions that rare *IFIH1* variants previously associated with type 1 diabetes are loss of function variants, and experimental data showing an important role of IFIH1 in antiviral immunity to enteroviruses, out data suggests that these variants have no or just minor impact on the frequency and duration of gut infection with enterovirus in healthy infants. Its effect on the risk of diabetes is likely to lie elsewhere in the pathogenic process than in the modification of gut infection frequency.

## Supporting Information

Table S1 **The effect of** ***IFIH1*** **genotypes on different measures of enterovirus infection in longitudinal fecal samples.**
(DOC)
