# Supplementary material for: Polymorphisms in the Innate Immune IFIH1 Gene, Frequency of Enterovirus in Monthly Fecal Samples during Infancy, and Islet Autoimmunity
Source: PLoS One. 2011 Nov 14;6(11):e27781. doi: 10.1371/journal.pone.0027781 (PMC3215739; doi:10.1371/journal.pone.0027781)
Supplement: Table S1 — The effect of IFIH1 genotypes on different measures of enterovirus infection in longitudinal fecal samples. (DOC) [file pone.0027781.s001.doc]

**Table S1.** The effect of *IFIH1* genotypes on different measures of enterovirus infection in longitudinal fecal samples

|  |  | **Infection episodes, N=7,494 (796 children)‡** | | | **Prolonged infections, N=7,034 (792 children)§** | | |
| --- | --- | --- | --- | --- | --- | --- | --- |
|  | **Genotypes** | **Frequency %** |  |  | **Frequency %** |  |  |
| ***IFIH1* SNPs*†** | **(n children)** | **(n samples/n total)** | **OR (95% CI)** | ***P*-value** | **(n samples/n total)** | **OR (95% CI)** | ***P*-value** |
| rs35337543 | GG (788) | 9.0 (668/7382) | 1.0 (ref) |  | 3.1 (211/6925) | 1.0 (ref) |  |
|  | CG (8) | 5.4 (6/112) | 0.6 (0.2-1.3) | 0.2 | 2.8 (3/109) | 0.9 (0.3-3.0) | 0.9 |
| rs35744605 | GG (786) | 9.0 (665/7401) | 1.0 (ref) |  | 3.1 (314/6950) | 1.0 (ref) |  |
|  | GT (10) | 9.7 (9/93) | 1.1 (0.5-2.3) |  | 0 (0/84) | n.a. |  |
| rs35667974 | AA (752) | 9.0 (641/7091) | 1.0 (ref) |  | 3.0 (200/6650) | 1.0 (ref |  |
|  | AG (44) | 8.2 (33/403) | 0.9 (0.6-1.3) | 0.6 | 3.7 (14/384) | 1.2 (0.7-2.1) | 0.7 |
| rs35732034 | GG (788) | 8.9 (664/7433) | 1.0 (ref) |  | 3.0 (210/6979) | 1.0 (ref) |  |
|  | GA (8) | 16.4 (10/61) | 2.0 (1.0-4.1) | 0.06 | 7.3 (4/55) | 2.5 (0.9-7.3) | 0.09 |
| rs1990760 | CC (135) | 7.7 (92/1191) | 1.0 (ref) | 0.4║ | 2.3 (26/1125) | 1.0 (ref) | 1.0║ |
|  | CT ( 386) | 9.4 (338/3601) | 1.2 (1.0-1.6) |  | 3.2 (109/3372) | 1.4 (0.9-2.2) |  |
|  | TT (275) | 9.0 (244/2702) | 1.2 (0.9-1.5) |  | 3.1 (79/2537) | 1.4 (0.9-2.1) |  |

SNP, single nucleotide polymorphism; OR, odds ratio; CI, confidence interval. *Location of *IFIH1* SNPs: rs35337543 (intron 18, 1641+1, G>C), rs35744605 (exon 10, E627X, G>T), rs35667974 (exon 14, I923V, A>G), rs35732034 (intron 14, 2807+1, G>A), rs1990760 (exon 14, A946T, T >C). †Reports of functional effects associated with *IFIH1* SNPs: rs35337543 and rs35732034 influences on a putative splice site, rs35744605 is associated with loss of function (ATPase activity, dsRNA binding, truncation of protein), rs35667974 is associated with loss of function (ATPase activity, dsRNA binding), and rs1990760 is not associated with loss of function (reviewed in [29]). ‡Excluding consecutively positive samples that may have been part of the same infectious episode as in the previous positive sample. §Excluding consecutively positive samples that may have been part of the same prolonged infection episode as in the previous positive sample and excluding infections that are not prolonged infections. ║Test for trend (1 d.f.).
